# Decision model of public opinion risk in campus social network based on hybrid dynamic deletion and shortest path algorithm

**DOI:** 10.1371/journal.pone.0310894

**Published:** 2024-11-18

**Authors:** Nan Xu, Yifeng Wang

**Affiliations:** School of Economics and Management, Xidian University, Xi’an, China; SASTRA Deemed University: Shanmugha Arts Science Technology and Research Academy, INDIA

## Abstract

Aiming at the problem that traditional network public opinion monitoring and searching are inefficient and can easily cause resource waste, the study firstly, through the dynamic deletion-shortest path algorithm to classify network text, and on this basis, innovatively constructs a text sentiment classification model based on the variant of convolutional neural network and recurrent neural network, and secondly, uses attention mechanism to classify the model. improvement of the classification model by using the attention mechanism. The research results show that the average precision rate, recall rate, and F-value of the dynamic deletion-shortest path algorithm are 97.30%, 79.55%, and 87.53%, and the classification speed is 397 *KB*/*s*, which is better than the traditional shortest path algorithm. In the classification effect measurement of long text, the accuracy and F-value of the recurrent neural network variant model are above 84%, and the accuracy of the text sentiment classification model with the introduction of the attention mechanism is improved by 3.89% compared to the pre-improvement period. In summary, the dynamic deletion-shortest path algorithm proposed in the study and the sentiment classification model with the introduction of the attention mechanism have superior performance and can provide certain application value for campus social network opinion risk decision-making.

## 1. Introduction

As the Internet’s product, social software not only meets people’s communication needs, but also provides a platform for students to freely express their emotions [1]. Most universities have their own social networking software, where students can exchange learning experiences and growth insights, making it an important place for students to engage in emotional communication [2]. Due to the fact that most social media apps fail to implement real name internet access, most students are easily misled by false and violent information, resulting in blurred concepts of right and wrong, and thus disrupting the harmony and stability of the campus [3,4]. To maintain the green environment of campus Internet culture, it is very important to establish the Internet public opinion risk decision making (IPORDM) mechanism. Traditional IPORDM mainly uses search engines to manually search for Internet public opinions (IPOs). These search results often contain a large amount of invalid information, and the search process is time-consuming and laborious, with low search efficiency [5,6]. Research needs to use computer technology to automatically collect, process, and process IPO information, and assist university managers in collecting and organizing IPOs. This can meet the needs of university management work, improve the real-time performance of IPO monitoring and formulating risk decision making (RDM) effectiveness. The key technologies of IPORDM based on computer technology include topic crawler technology, text segmentation technology, and sentiment classification technology. Text segmentation technology is the foundation of information processing, and sentiment classification technology is an important prerequisite for sentiment analysis of IPO texts. Shortest Path (SP) algorithm can eliminate the semantic ambiguity of text. In the experiment, it was applied to the design of text segmentation algorithms and Dynamic Deletion (DD) was introduced to improve SP algorithm, resulting in DD algorithm. On this basis, the study also integrated Convolutional Neural Network (CNN), Bidirectional Long Short Term Memory (BLSTM), and Bidirectional Gated Recurrent Unit (BGRU) to construct a Hybrid Convolutional and Recurrent Neural Network (HCRNN) model. Simultaneously, using Attention Mechanism (AM) to improve HCRNN, HCRNN-AM was obtained for sentiment classification of IPO texts [7]. The research aims to improve the efficiency of word segmentation of online text and SPeed of Sentiment analysis, so as to improve the real-time and accuracy of IPORDM formulation of campus social networking. The innovation points of the research mainly include the following two points. Firstly, the introduction of DD improves SP algorithm to obtain DD algorithm. Secondly, aiming at the problem of different length of network text, the research uses CNN to extract local features of text, and BLSTM and BGRU to extract text context information to improve sentiment analyzing classifying effect. The research structure mainly includes four parts. The first is a review of relevant research results. Next is the construction of HCRNN-AM based on DD algorithm in campus social IPORDM. The third part is to verify the feasibility of the model proposed by the research institute. Finally, there is a summary of the research.

## 2. Related works

SP refers to the path that leads from a vertex to another vertex along the edge of the graph, with the minimum sum of weights on each edge. SP problem is a classic algorithmic problem in graph theory research, which is widely applied in various fields and has been deeply explored by many scholars. Ardizzoni et al. proposed a bounded acceleration SP algorithm under the constraint that the vehicle must meet the maximum and minimum acceleration and speed constraints. This algorithm aims to find the shortest time path between two moving vehicle positions. These results confirm that the bounded accelerated SP algorithm performs well [8]. Jiang et al. addressed the issue of low accuracy in multi graph matching by using offline batch processing mode and online settings to express the multi graph matching problem as searching for all paired SPs and single source SPs on the hypergraph. And they used the Freudian algorithm and SP fast algorithm for solving. These results confirm that the Freudian algorithm and SP fast algorithm can effectively find the optimal path [9]. Klančar et al. designed a hybrid path algorithm to solve smooth path planning problems, which combines discrete network based E search with continuous Bernstein Bezier motion primitives and is a two-stage algorithm. Through simulation, it has been confirmed that the hybrid path algorithm not only generates collision safe and smooth paths that are close to the optimal space, but also ensures curvature continuity [10]. Considering the traffic safety of urban navigation, Jiang S proposed an improved Tabu search algorithm based on data-driven dynamic SP problem. The improved Tabu search algorithm has different initial solutions, and adopts subgraph and adaptive insertion technology as the acceleration strategy to improve the computational efficiency of dynamic SP problems. These simulation results confirm the computational performance and solution quality of the improved Tabu search algorithm [11]. Owais and Shahin designed a heuristic algorithm to solve the problem of locating traffic sensors on a completely separated traffic network with all start/end node pairs. This heuristic algorithm mobilizes the dual formula and proposes an SP based column generation method that will generate as many paths as needed to avoid the challenging task of enumerating complete paths. These results confirm that the heuristic algorithm has high scalability [12].

Text classification technology utilizes predefined text categories to guide the learning of new test texts, thereby determining the category of new texts. In IPORDM, classifying network texts is a necessary prerequisite. Xu et al. proposed a selective ensemble model on the foundation of CNN to make the classification ability and robustness of text improved. At the same time, they utilize different sample subsets and different granularity convolutional kernels to enhance the diversity of selective ensemble models. These simulation results confirm the effectiveness and universality of the selective ensemble model based on CNN [13]. Li et al. designed a flexible short text data stream classification method based on Long Short-Term Memory (LSTM) ensemble network to address the problem of data sparsity and concept drive (CD) in short text data streams on social networks. This classification method first utilizes pre trained embedding models and CNN’s short text embedding based on external resources to address SParsity of text data. Then they will develop a distributed LSTM integrated network for the classification of short text data streams. Finally, the CD factor was introduced in the experiment to adapt to the CD caused by changes in data distribution. These results confirm that the classification method proposed in the study can effectively classify short text data streams while adapting to CD [14]. Tan et al. proposed a dynamic embedded projection gate CNN for multi class and multi label text classification to improve the accuracy of text classification. The dynamic embedded projection gate CNN mainly converts and carries word information through the use of gating units and quick connections. This can control that multiple context information is merged into a specific position in the text word embedding matrix. These simulation results confirm that the dynamic embedded projection gate CNN is superior to other similar algorithms [15]. E-sic system has issued too many requests, making it difficult to respond to them. Paiva et al. created an automatic classifier based on CNN and LSTM structures. Error function used by the automatic classifier is cross entropy, and the measurement used for its evaluation result is receiver operating characteristic and accuracy. These simulation results confirm that the automatic classifier can effectively classify Portuguese text [16]. Guo et al. designed a supervised contrastive learning algorithm with term weighting to improve the effectiveness of Chinese text classification. This supervised contrastive learning algorithm mainly optimizes the data expansion process of Chinese text by calculating the weight of terms, and incorporates the transformed features into a time convolutional network for feature representation. These results confirm that supervised contrastive learning algorithms with term weighting have higher classification speed and accuracy compared to other Chinese text classification algorithms [17].

In summary, there are many research achievements on SP problem and text classification technology, but there are few studies that combine SP problem with text segmentation technology. Text classification technology also mostly uses CNN to process data, which has low computational efficiency in online text sentiment classification and does not utilize the development of IPORDM. In response to the above issues, the study first utilizes DD algorithm to segment network text, then fuses CNN, BLSTM, and BGRU structures to obtain HCRNN, and introduces AM to improve HCRNN to obtain HCRNN-AM.

## 3. HCRNN-AM construction based on DD algorithm in campus social IPORDM

The social platforms within universities serve as a shared spiritual belonging for students, enriching their extracurricular life and strengthening communication and interaction with others. Due to the strong anonymity of most social media platforms, they can easily become tools for illegal individuals to make false statements. Therefore, establishing a social IPORDM mechanism is crucial. This chapter focuses on the design of DD algorithm based on DD and SP algorithms and the construction of text sentiment classification model based on HCRNN.

### 3.1 Design of DD algorithm based on DD and SP algorithms

**Fig 1:**
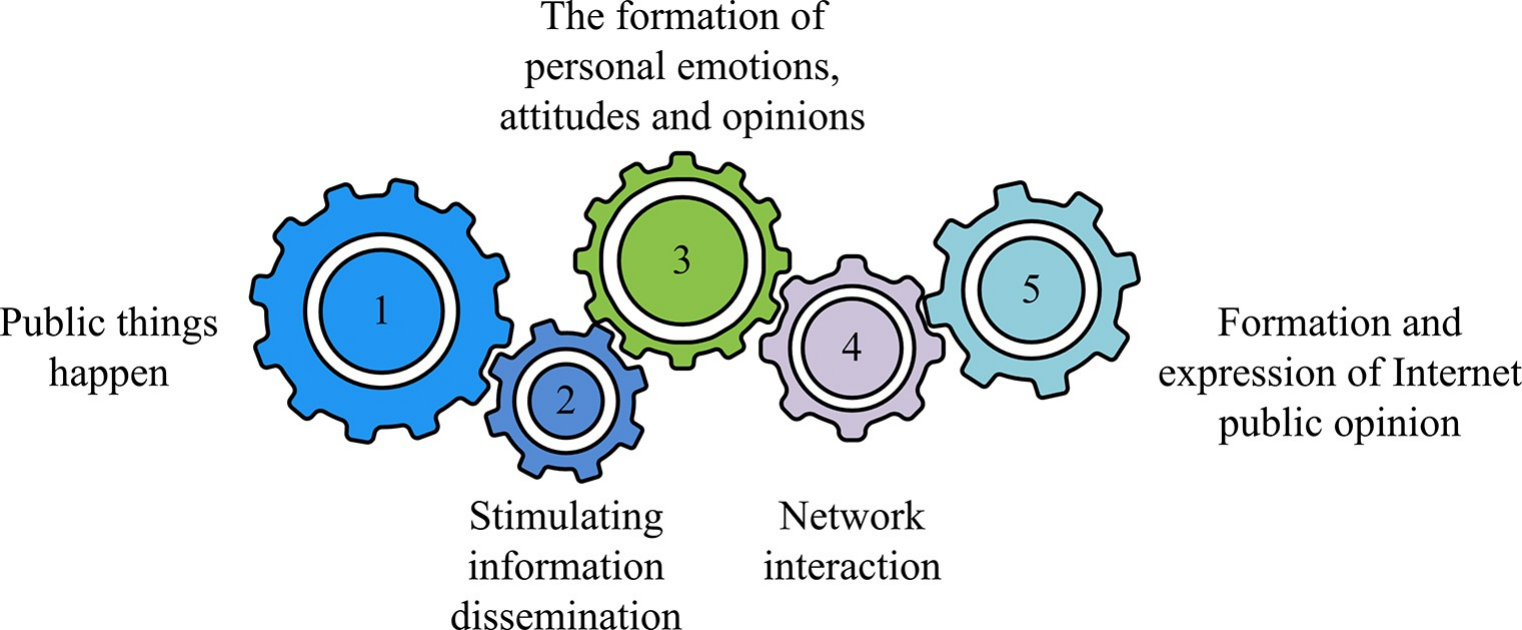
Schematic diagram of the formation process of network public opinion.

IPO refers to the popular online public opinion that has different views on real and virtual social issues on the internet. They are influential and biased opinions and opinions of the public on certain hot topics and focal issues in real life that are disseminated through the internet. Among them, the internet, events, netizens, emotions, communication and interaction, and influence are the six elements that make up an IPO. Fig 1 shows the formation of an IPO.

The formation of IPO in Fig 1 is a linear process, with each stage interconnected. Due to the open anonymity and strong interactivity of campus social networks, when some posts intentionally exaggerate or maliciously distort facts, once the posts are intentionally guided and spread, it is very easy to cause conflicts. The release of some useless and false information can easily lead to confusion in college students’ concepts of right and wrong, and confusion in their understanding of values, outlook on life, and ideology. Therefore, monitoring campus social IPOs is a necessary measure. Text segmentation, as the foundation of IPORDM, is the process of dividing a text sequence into individual words and transforming consecutive word sequences into composite word sequences according to certain specifications. The existing segmentation algorithms are mainly divided into dictionary based segmentation methods, statistical segmentation methods, and understanding based segmentation methods. The premise of a dictionary based word segmentation method is to have a dictionary that includes all vocabulary as much as possible, and then scan the sentences to be segmented according to certain rules to match the entries in the dictionary. If the match is successful, the entry will be segmented, otherwise other processing will be performed. The forward matching algorithm, as an algorithm in string based word segmentation, matches from left to right based on key vocabulary. The forward matching algorithm first requires corpus preprocessing of the processed text. Then, it is necessary to read it into the dictionary to establish an index and use a forward matching algorithm for preliminary distribution. Then, rule based methods can be further used to eliminate ambiguity in the segmentation results, thereby obtaining the final segmentation result. Although dictionary based word segmentation methods are not efficient and difficult to handle ambiguity, their implementation is simple, so most word segmentation algorithms are still based on dictionary based word segmentation methods. On this basis, SP algorithm is proposed to match the set of all possible words in the text string according to the order in the dictionary, and take all words as a node to construct a directed acyclic graph. In this directed acyclic graph, among all the paths from a starting point to an ending point, the research needs to find all the path values from each node to the source node. Each path value corresponds to a path set as its corresponding segmentation result set. In SP algorithm, the best first search algorithm is the most representative one. As a heuristic algorithm, it is very effective in solving the shortest path in static road networks in Eq (1).


f(n)=g(n)+h(n) (1)

**Fig 2:**
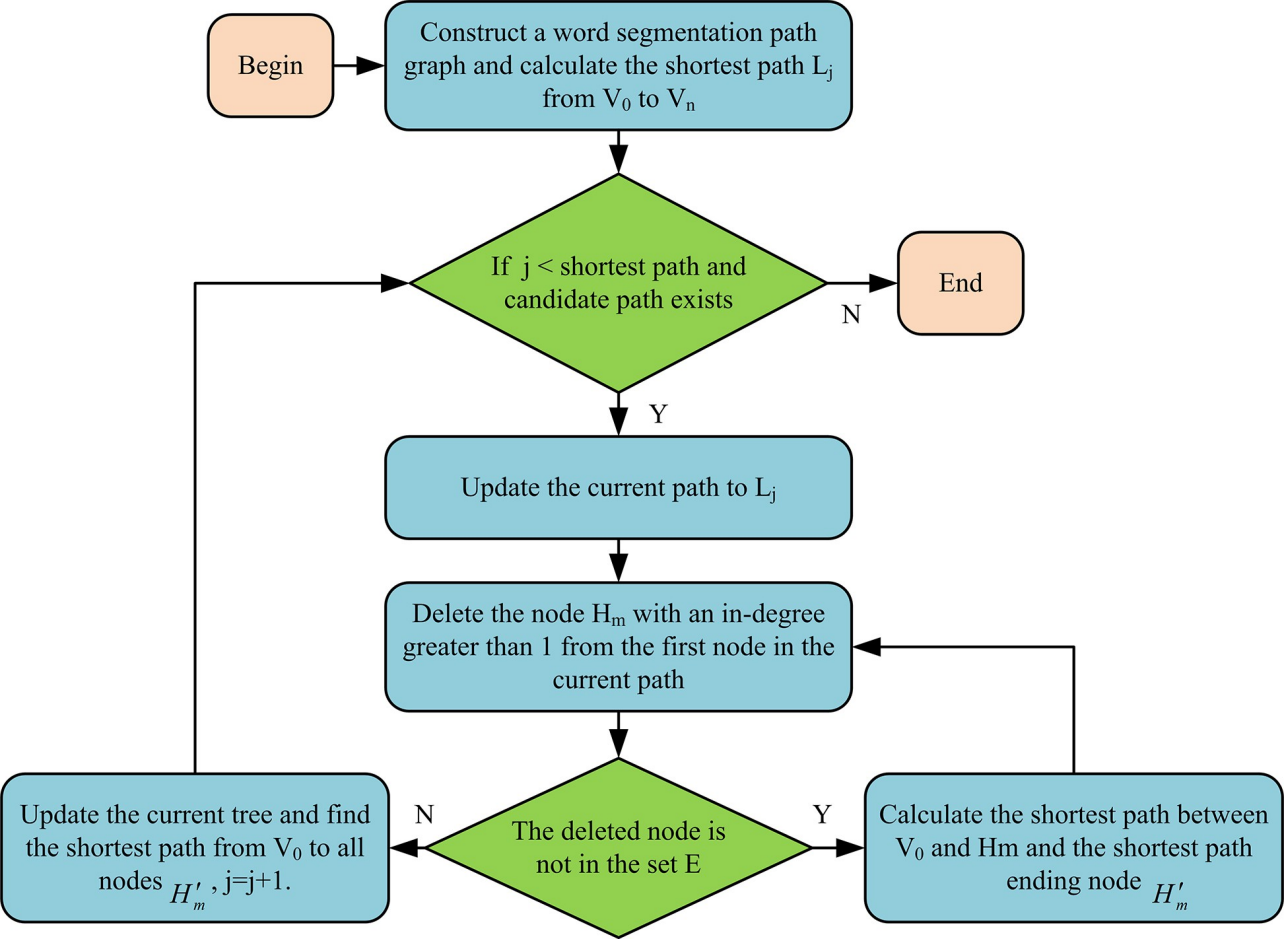
Schematic diagram of DD algorithm flow.

In Eq (1), *f*(*n*) is the evaluation function of node *n* from the initial point to the target point. *g*(*n*) is the actual cost from the initial node to node *n* in the state space. *h*(*n*) is the estimated cost of the optimal path from node *n* to the target node. The solution of SP algorithm needs to calculate all the path values from the starting point to the end point in directed acyclic graph, which requires a large amount of calculation, resulting in low segmentation efficiency. To improve the word segmentation efficiency of SP algorithm, DD algorithm is studied by citing DD for improvement. DD is the creation of an SP tree update queue that saves all descendants of nodes that will be deleted to that queue. The node that needs to be deleted and all its descendants have been removed from the original SP tree. The node with the shortest distance from the root node was selected from the queue and updated. The updated nodes are no longer inserted into the queue, thereby reducing the number of node updates. Fig 2 shows the process of DD algorithm.

In Fig 2, the word segmentation path was first constructed based on SP algorithm, and SP *L*_*j*_ from a starting node *V*_0_ to an ending node *V*_*n*_ was calculated. If *j* is less than the number of SPs and the candidate path exists, the current path *L* to *L*_*j*_ is updated. Then, the first node *H*_*m*_ in the current path with a starting degree greater than 1 is deleted. If the descendant node of the deleted node is not in the set *E*, the corresponding SP from *V*_0_ to *H*_*m*_ is calculated, and the end node of the path is denoted as $H_{m}^{\prime }$. If the descendants of the deleted node are in *E*, then *H*_*m*_ and all its descendants are removed from *E*. This research also needs to continue updating the current path tree to obtain SP from *V*_0_ to all nodes $H_{m}^{\prime }$. At this point, *j* = *j*+1, and the above steps are repeated.

### 3.2 Design of CNN and RNN variants

Due to the openness and convenience of campus social networks, students often use the internet to express their opinions, which often contain rich personal emotions. How to perform emotional analysis from these online texts is a research hotspot in IPORDM. Emotional analysis is the determination of SPeaker or author’s attitude towards a specific topic, where attitude refers to the author’s viewpoint on a certain matter. The important premise of emotional analysis is emotional classification, which is the judgment of the author’s emotional attitude. Considering the issue of inconsistent text length on campus social networks, a HCRNN fusion model combining CNN and Recurrent Neural Network (RNN) variants was proposed for sentiment classification tasks [18,19]. As a mainstream algorithm in deep learning, CNN can be used to process data with similar network structures and has good classification performance in short texts. CNN is composed of a series of convolutional, activation, pooling, and fully connected layers. The convolutional layer is the core of CNN, which can extract image features. Multiple convolutional calculations can effectively reduce the complexity and training parameters of the algorithm. Eq (2) is the parameter of the convolutional layer.


$$x_{j}^{l}=f(\sum_{i\in M_{j}}x_{i}^{l-1}*Kernel_{ij}^{l}+b^{l})$$ (2)

**Fig 3:**
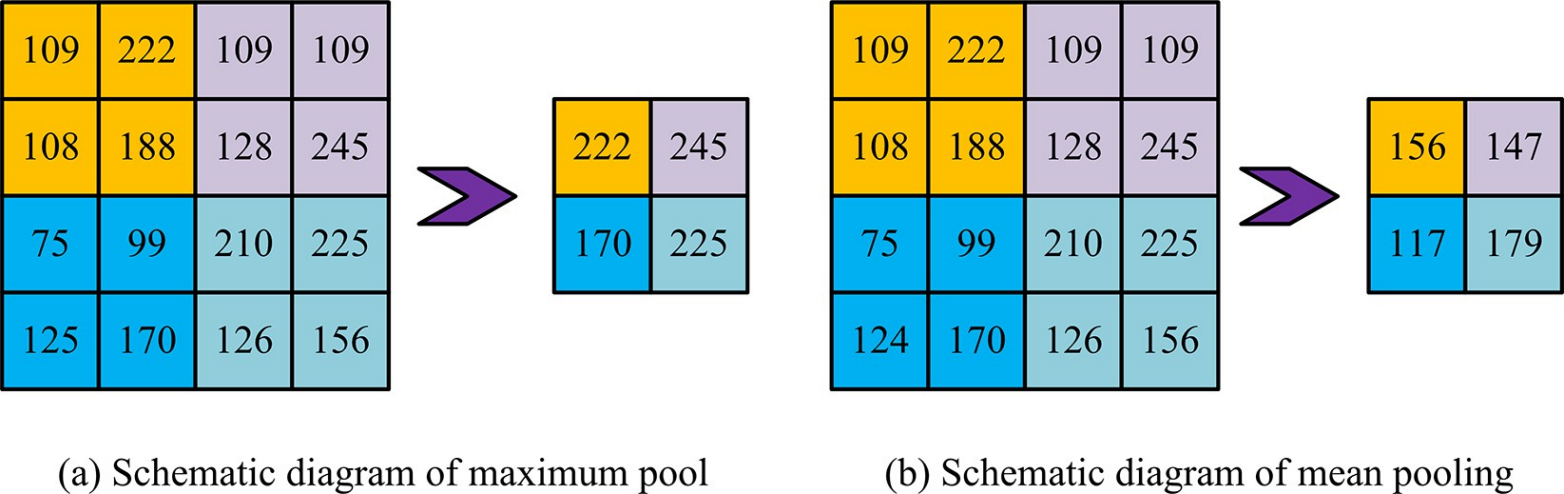
Pooling.

In Eq (2), $x_{j}^{l}$ is the *j*-th neuron in the *l*-th layer. *Kernel*, *, and *f*(•) represent convolutional kernel, convolutional operation, and nonlinear excitation function, respectively. *a* and *M*_*j*_ represent the bias term and the number of inputs to the *j*-th neuron, respectively. The pooling layer can reduce the dimensionality of feature maps and reduce the risk of overfitting. The commonly used pooling operations are mean pooling and maximum pooling in Fig 3.

Fig 3A shows maximum pooling. Fig 3B shows mean pooling. Maximum pooling preserves each sliding window’s maximum value, while mean pooling preserves all sliding windows’ average value. The excitation layer introduces nonlinear features into the neural network, enabling it to approximate any nonlinear function, thereby effectively improving the feature expression ability of CNN. Eqs (3) to (5) are commonly used Activation function.


$$f_{S}(x)=\frac{1}{1+e^{-x}}$$ (3)

In Eq (3), *f*_*s*_(*x*) represents Sigmoid function, with a range of (0,1) corresponding to the probability range (0,1). Eq (4) isTanh function.


$$f_{T}(x)=\frac{e^{x}-e^{-x}}{e^{x}+e^{-x}}$$ (4)

The output of Eq (4) is centered around 0, which can achieve the effect of data centralization. Eq (5) is ReLu function.


$$f_{R}(x)=\{\begin{array}{l} x,x>0\\ 0,x<0 \end{array}$$ (5)

Due to the saturation zone between Sigmoid and Tanh functions, the gradient is prone to vanishing during backpropagation. ReLu function exists to solve the above problem, with a positive activation value derivative of 1, which can effectively avoid the phenomenon of gradient vanishing. The fully connected layer’s function is to integrate input text features and map the feature maps generated by the convolutional layer into fixed length feature vectors, thus completing the text classification task in Eq (6).


$$x_{j}^{l}=f(\sum_{i\in M_{j}}x_{i}^{l-1}*w_{i,j}+b_{j})$$ (6)

**Fig 4:**
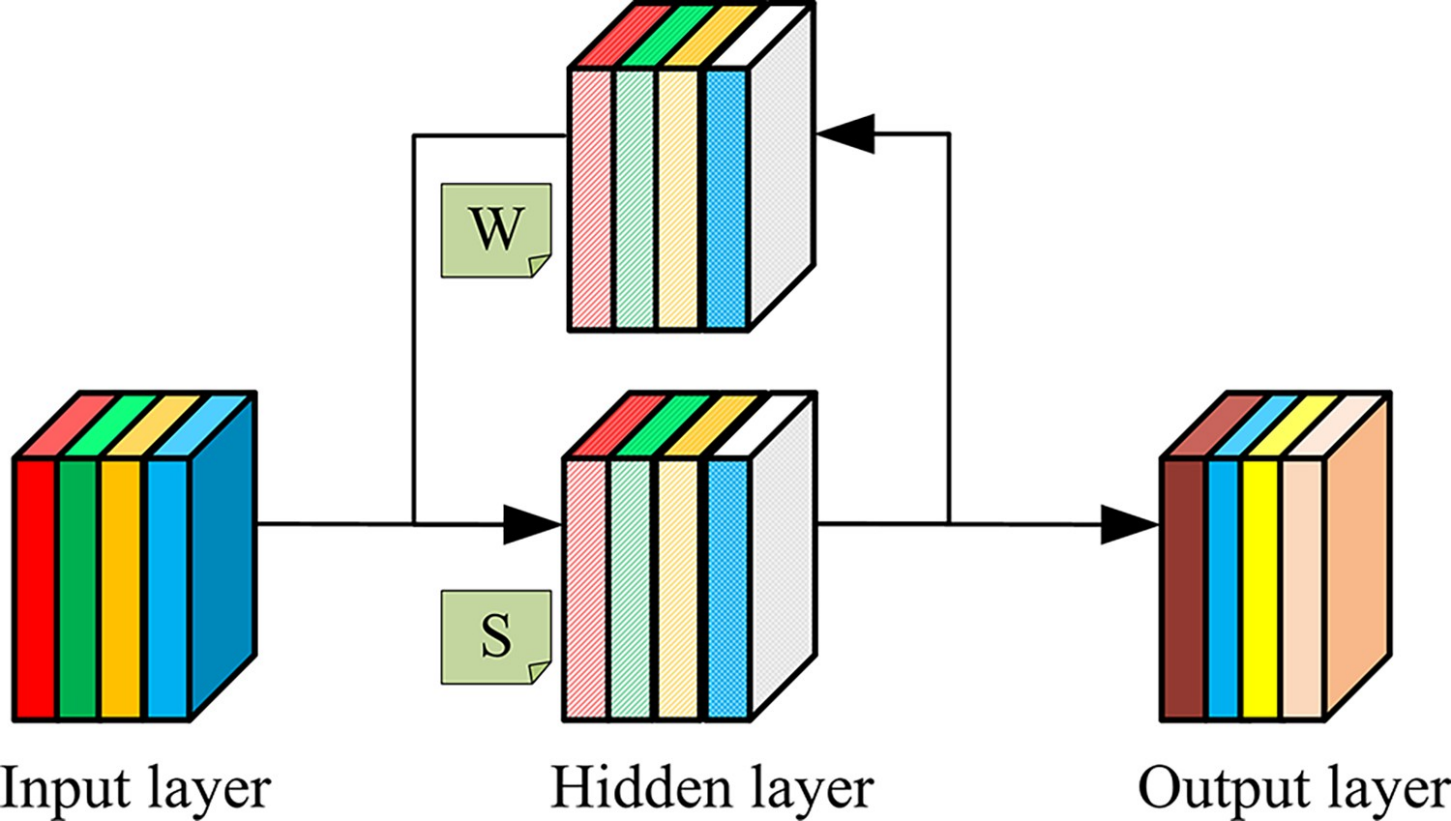
Schematic diagram of RNN.

In Eq (6), $x_{j}^{l}$ is the fully connected layer’s output neuron. *w*_*i*,*j*_ and *b*_*j*_ represent the weights and offsets between neurons, respectively. RNN is mainly used to process sequence data, and its essence is to have temporal memory like humans. In theory, it can input sequences of any length. However, in practical applications, it can only utilize effective step sizes. Fig 4 shows the structure of RNN.

*S* in the hidden layer in Fig 4 depends not only on the current input *X*, but also on the previous value of that layer. For moment *t*, Eq (7) is the calculation of RNN.


$$\left\{ \begin{array}{l} O_{t}=g(V_{S_{t}})\\ S_{t}=f(U_{X_{t}}+W_{S_{t-1}}) \end{array}\right.$$ (7)

**Fig 5:**
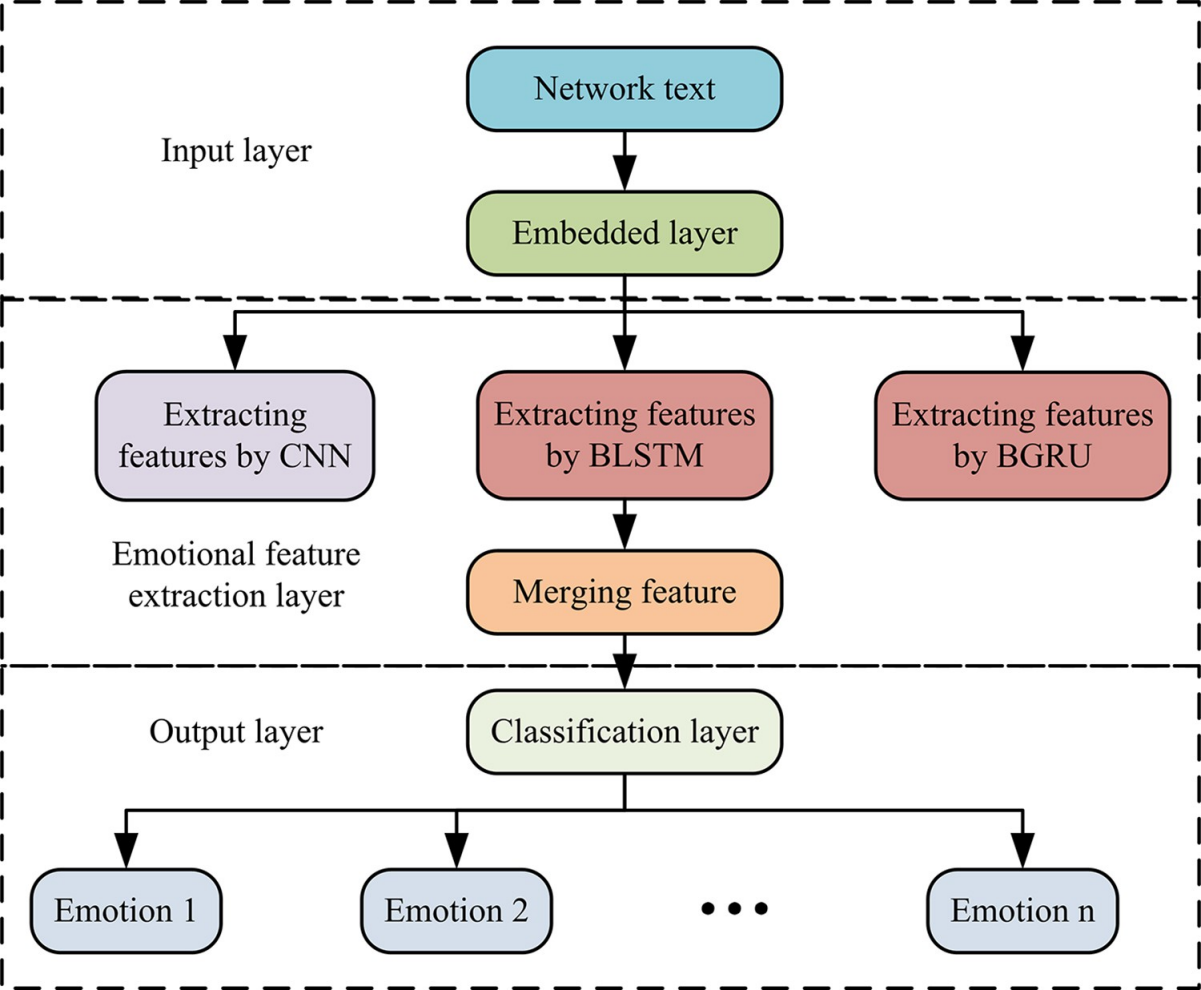
Schematic diagram of overall framework of HCRNN model.

In Eq (7), *O*_*t*_ is the output value at time *t*. *f*(*x*) and *g*(*x*) are activating functions. *U*, *V*, and *W* represent a weight of inputting layer to hidding layer, the weight of hidding layer to outputting layer, and a previous value of hidding layer as inputting weight for this time. *X*_*t*_ and *S*_*t*_ represent the input values and hidden layer values at time *t*, respectively. Due to changes in applications and requirements, the RNN structure has become increasingly complex. LSTM and Gated Recurrent Unit (GRU), as variants of RNN, perform well in sentiment classification of long text on the network. Therefore, the study introduces BLSTM and BGRU to extract contextual features of network texts [20]. Therefore, Fig 5 shows the overall framework structure of HCRNN that integrates CNN and RNN variants.

HCRNN in Fig 5 is divided into an input layer, an emotion feature extraction layer, and an output layer. This research first needs to input text from campus social networks into the embedding layer to convert network text into word vectors. Then, CNN and RNN variants are used to extract local and contextual features of the text. And the extracted two types of features are combined to input into the classification layer, completing the emotional classification of the network text.

### 3.3 Construction of a text sentiment classification model based on HCRNN

The main function of the input layer in HCRNN is to convert network text on campus social networks into word vectors, which is performed within the embedding layer. This study utilizes DD algorithm to divide text *T* into *n* words, and its corresponding matrix is represented as *W*_*n*×*d*_. *d* is word vector dimension, so Eq (8) is the text matrix *W*.


$$W=v_{1}^{d}⊕v_{2}^{d}⊕\cdots ⊕v_{n}^{d}$$ (8)

In Eq (8), *v*_*i*_∈*R*^*d*^ is the word vector of the *i*-th word in text. ⊕ refers to series operation. The emotional feature extraction layer in HCRNN is mainly divided into CNN module and RNN variant module. The input of the CNN module is a text matrix *W* output by the embedding layer. To better extract emotional features from short texts on the internet, three parallel convolutional kernels with sizes *h*_1_×*d*, *h*_2_×*d*, and *h*_3_×*d* were used to slide the matrix *W* from top to bottom. Considering the context information of text, this study used padding to perform zero padding on the original input information during convolution operations, with a padding size of half the convolutional kernel’s height. After filling with 0, *W* yields $\bar{W}$ in Eq (9).


$$\bar{W}=\underset{\left[h/2\right]}{\underset{︸}{w_{0}⊕w_{0}\cdots ⊕w_{0}}}⊕W⊕\underset{\left[h/2\right]}{\underset{︸}{w_{0}⊕w_{0}\cdots ⊕w_{0}}}$$ (9)

In Eq (10), *w*_0_ is the 0 vector. 0 vector dimension is consistent with the word vector dimension. *h* refers to the height of the convolutional kernel. After $\bar{W}$ passing through the convolution kernel, the eigenvector *v* is ultimately obtained in Eq (10).


$$v={\left(v_{1},v_{2},\cdots ,v_{k}\right)}^{T}$$ (10)

**Fig 6:**
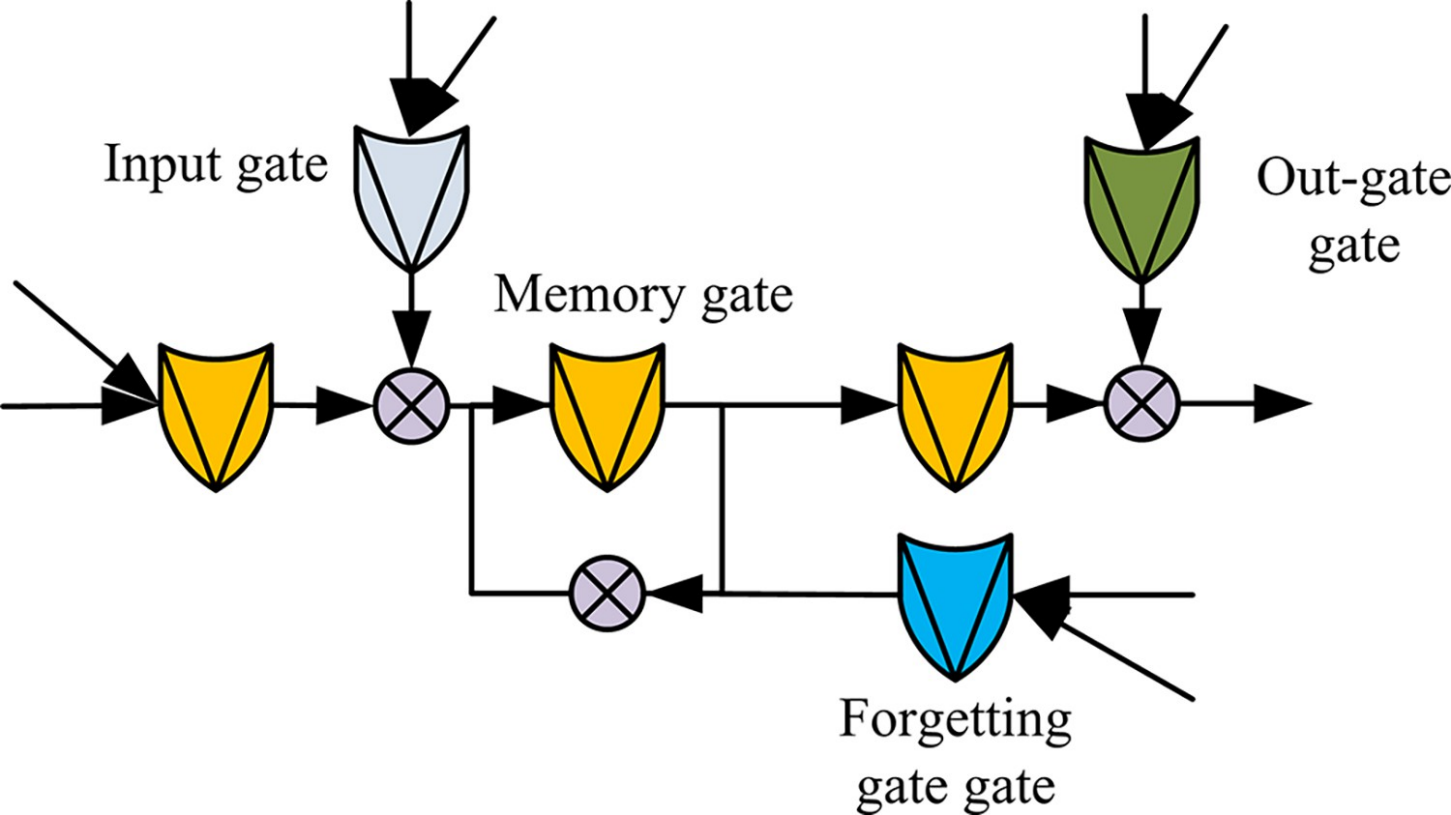
Schematic diagram of LSTM.

In Eq (11), *k* is the number of convolutional kernel slides. Due to the fact that network text is a type of temporal data and contexts are interdependent, the study involves extracting contextual features of network text through BLSTM and BGRU networks. BLSTM is composed of front and rear LSTMs, and the output is Co-determination determined by these two LSTMs. Fig 6 shows the model structure of LSTM.

Compared with ordinary RNN, LSTM model in Fig 6 adds input gate, output gate, forgetting gate, and memory gate mechanisms. The reason for using BLSTM in research is that it can better capture the bidirectional semantic dependencies in sentences and can capture the interaction relationships between words at a finer granularity. Eq (11) represents the BLSTM output value *h*_*t*_ at time *t*.


$$\left\{ \begin{array}{l} h_{t}=concat(h_{Lt},h_{Rt})\\ h_{Lt}=LSTM_{L}(v_{t},h_{L(t-1)})\\ h_{Rt}=LSTM_{R}(v_{t},h_{R(t-1)}) \end{array}\right.$$ (11)

In Eq (11), *h*_*Lt*_ and *h*_*Rt*_ are the outputs of the backpropagation and forward propagation network time *t*. *LSTM*_*L*_ and *LSTM*_*R*_ represent LSTM forward and backward propagation, respectively. *v*_*t*_ is the input word vector at time *t*. After passing through BLSTM layer, Eq (12) represents the output *H* of HCRNN.


$$H=[\begin{array}{c} h_{L1},h_{R1}\\ h_{L2},h_{R2}\\ \vdots \\ h_{Lc},h_{Rc} \end{array}]$$ (12)

**Fig 7:**
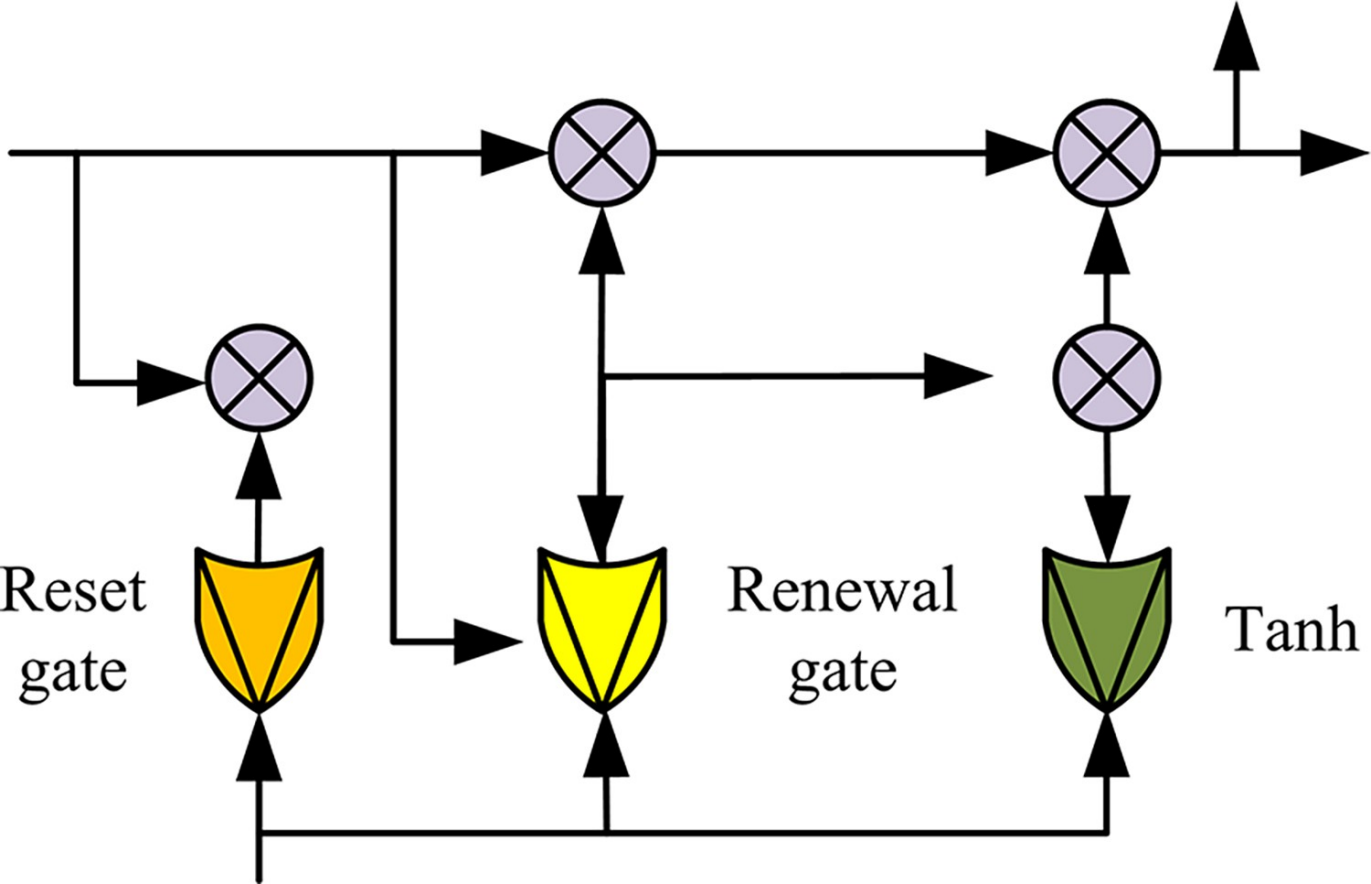
Schematic diagram of GRU model structure.

In Eq (12), *c* is the number of neurons in BLSTM. To extract deeper contextual features from network text context, BGRU was designed in the next layer of bidirectional memory neural networks. Compared to bidirectional memory neural networks, BGRU is relatively simple and has smaller parameters. BGRU and BLSTM are composed in the same way. They are composed of forward and backward GRUs. The output is determined by these two GRUs. Fig 7 shows the structure of GRU.

In Fig 7, a single GRU contains a reset gate and an update gate. In BGRU, Eq (13) represents the output value *g*_*t*_ at time *t*.


$$\left\{ \begin{array}{l} g_{t}=w_{Lt}g_{Lt}+w_{Rt}g_{Rt}\\ g_{Lt}=GRU_{L}(h_{t},g_{L(t-1)})\\ g_{Rt}=GRU_{R}(h_{t},g_{R(t-1)}) \end{array}\right.$$ (13)

In Eq (14), *GRU*_*L*_ and *GRU*_*R*_ represent GRU backward and forward propagation, respectively. *w*_*Lt*_ and *w*_*Rt*_ respectively represent the weight of the results obtained by the backward and forward GRU networks in the final results. So Eq (14) is the output *G* of BGRU.


$$G=[\begin{array}{c} g_{L1},g_{R1}\\ g_{L2},g_{R2}\\ \vdots \\ g_{Ls},g_{Rs} \end{array}]$$ (14)

In Eq (14), *s* is the number of neurons in BGRU network. In online texts, words with emotional colors have a significant impact on the final emotional classification in the text. To distinguish the degree of influence of different texts on the final sentiment classification of texts, this study introduced AM on the basis of HCRNN to obtain HCRNN-AM. The addition of AM can enable the model to allocate more attention to content that has a significant impact on classification results, thereby reducing the attention of unimportant content. It is usually added to the next layer of BGRU layer, with the input source being the output of BGRU.

## 4. Analysis of HCRNN-AM results based on DD algorithm

In order to verify the effectiveness and feasibility of the algorithms and models proposed in the study, the study sets up several control groups for experiments, and the chapter focuses on the performance analysis of the DD-SP algorithm based on DD and SP algorithms and the performance analysis of the HCRNN-AM model with the addition of AM.

### 4.1 Performance analysis of DD algorithm

To verify DD algorithm’s effectiveness, comparative experiments are conducted using Word Segmentation Based on Dictionary (WSD), Word Segmentation Based On Statistics (WSS), and SP algorithms, respectively. All experimental data comes from network data collected by crawler tools. This research sets the experimental data into 8 groups, with each test sample text of about 30M and a total of 300 test texts. The performance indicators are precision, recall, F-value, and word segmentation speed.

**Fig 8:**
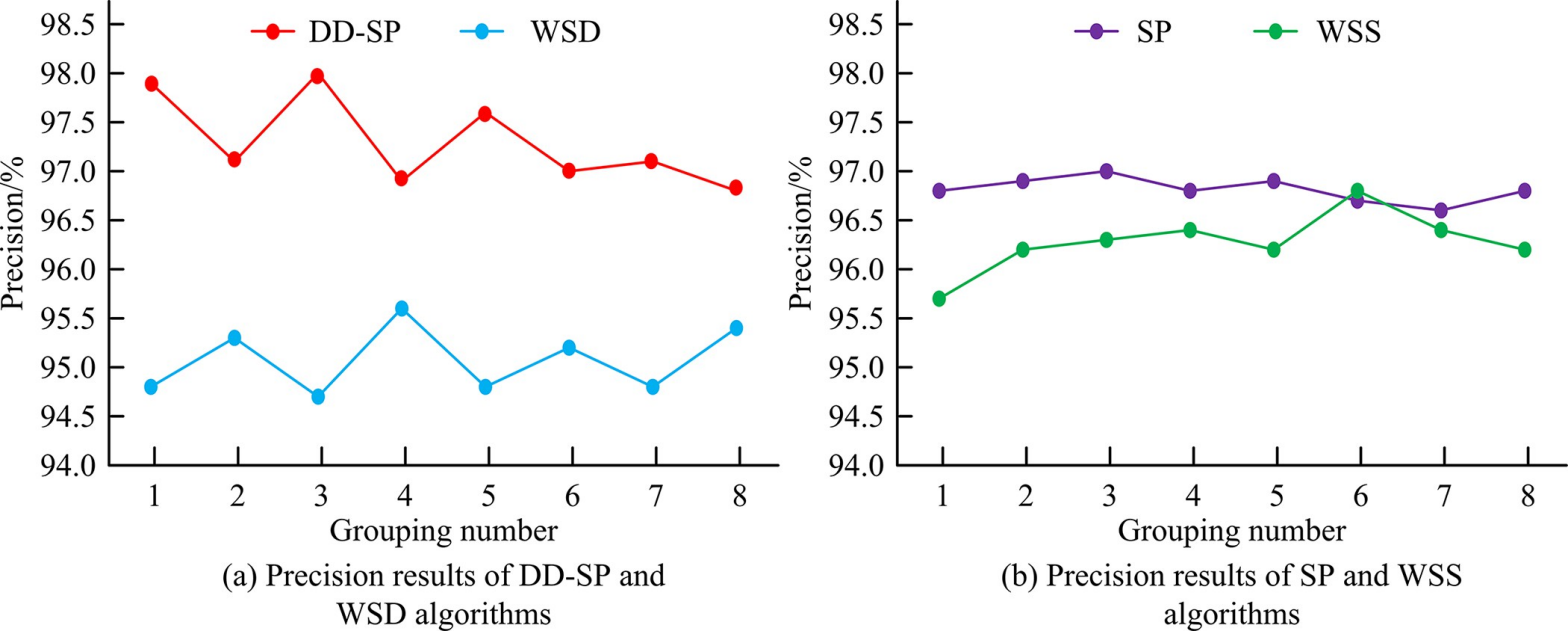
Precision.

Fig 8A shows the precision results of DD and WSD. Fig 8B shows the precision results of SP and WSS. In the precision experiment of the first group, DD was 97.91%, SP was 96.78%, WSD was 94.79%, and WSS was 95.72%. In the precision experiment of group 5, DD was 97.58%, SP, WSD, and WSS were 96.92%, 94.77%, and 96.19%, respectively. In the precision experiment of group 8, DD, SP, WSD, and WSS were 96.80%, 96.74%, 95.43%, and 96.22%, respectively. According to Fig 8, the average precision of DD is 97.30%, which is higher than 96.81% of SP, 95.08% of WSD, and 96.28% of WSS.

Table 1 shows four algorithms’ recall results. In the recall rate experiment of the first group, DD was 79.11%, SP, WSD, and WSS were 78.02%, 78.59%, and 79.36%, respectively. In the recall rate experiment of group 5, DD was 79.95%, SP was 77.84%, WSD was 77.10%, and WSS was 77.47%. In the recall rate experiment of group 8, DD was 80.70%, higher than 75.83% of SP, 76.84% of WSD, and 77.74% of WSS. Based on the data in Table 1, the average recall rates of DD, SP, WSD, and WSS algorithms are 79.55%, 77.69%, 77.71%, and 77.76%, respectively. Due to the mutual constraints between algorithm’s precision and recall, when the precision is high, its recall is low. When the recall rate is high, its precision is low. To comprehensively reflect DD algorithm’s performance, the study utilizes the metric value F for overall performance evaluation.

**Table 1:** Recall results of four algorithms.

Algorithm category	1	2	3	4	5	6	7	8
DD	79.11%	78.82%	78.38%	80.26%	79.95%	79.86%	79.30%	80.70%
SP	78.02%	77.32%	77.97%	79.90%	77.84%	78.16%	76.48%	75.83%
WSD	78.59%	78.01%	78.33%	77.04%	77.10%	78.70%	77.10%	76.84%
WSS	79.36%	77.01%	76.59%	78.84%	77.47%	77.89%	77.11%	77.74%

**Fig 9:**
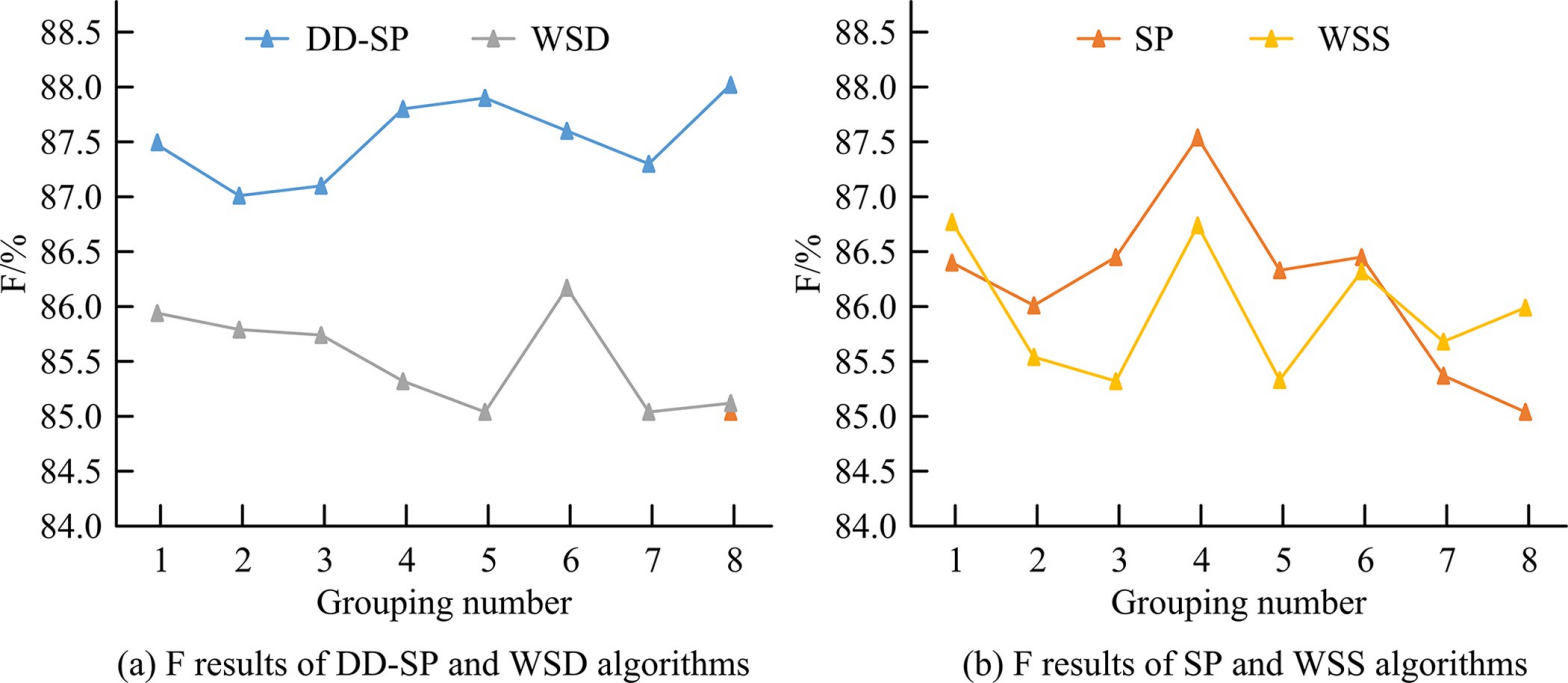
F-value.

Fig 9A shows DD and WSD algorithms’ F-value results. Fig 9B is SP and WSS algorithms’ F-value results. F-values of DD, SP, WSD, and WSS algorithms in the first group were 87.50%, 86.40%, 85.94%, and 86.77%, respectively. F-value of DD algorithm in group 5 is 87.91%, which is higher than the 89.89%, 86.33%, 85.04%, and 85.31% of SP, WSD, and WSS algorithms in the same group. For Group 8, F-value of DD is 88.07%, SP is 85.04%, WSD is 85.12%, and WSS is 85.99%. According to Fig 9, the average F-value of DD is 87.53%, while the average F-value of SP, WSD, and WSS algorithms is 86.17%, 85.46%, and 85.85%. Word segmentation speed refers to the number of text words processed per unit time. When word segmentation accuracy, recall, and F-value meet the requirements, word segmentation speed is another important indicator.

**Fig 10:**
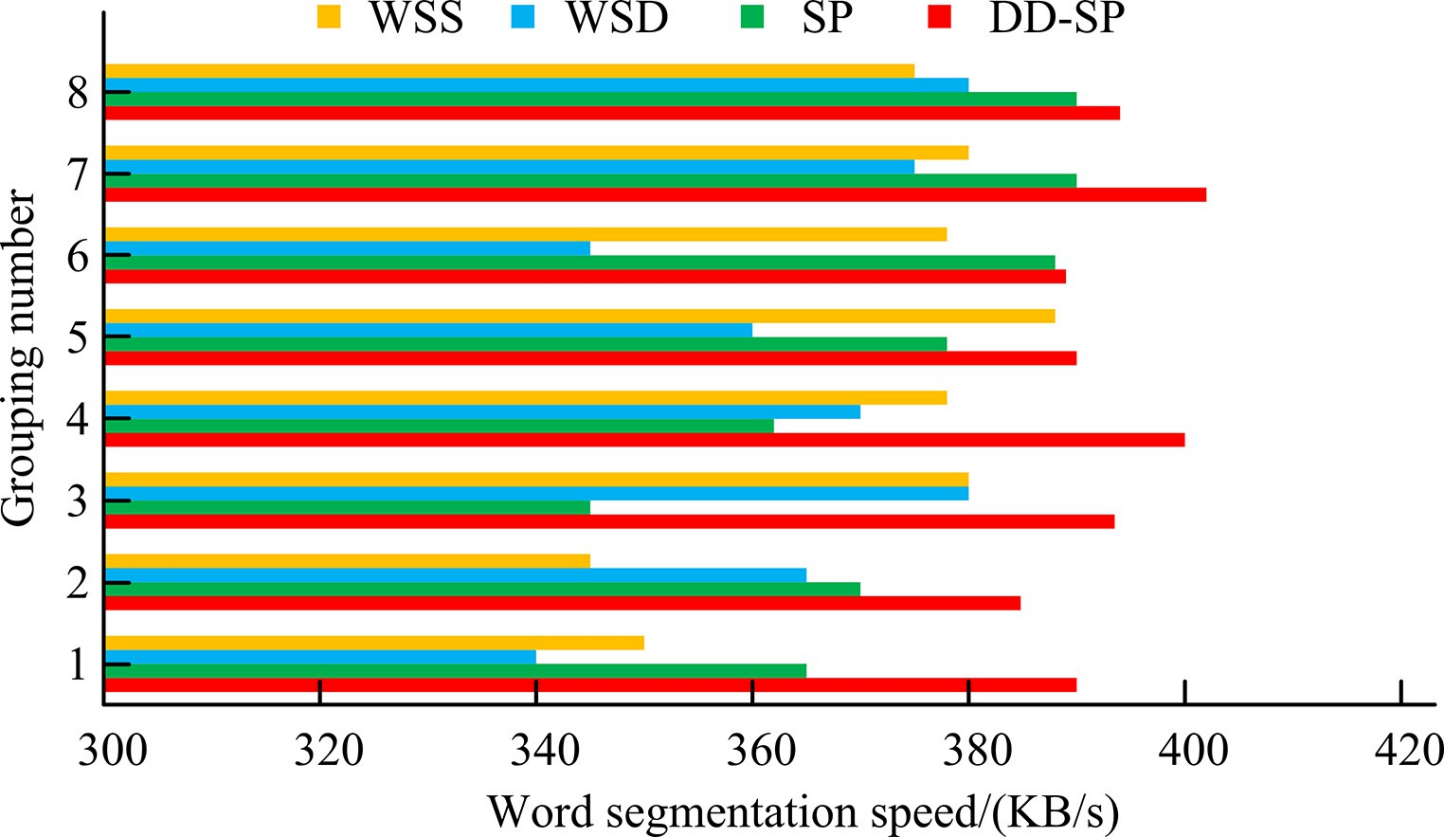
Word segmentation speed results of four algorithms.

Fig 10 shows the segmentation speed results of four algorithms. For the word segmentation speed of group 1, DD is 390 *KB*/*s*, SP is 365 *KB*/*s*, WSD is 340 *KB*/*s*, and WSS is 350 *KB*/*s*. In the word segmentation speed of Group 5, DD is 398 *KB*/*s*, while SP, WSD, and WSS algorithms are 378 *KB*/*s*, 360 *KB*/*s*, and 388 *KB*/*s*, respectively. In the word segmentation speed of Group 8, DD and SP algorithms are 394 *KB*/*s* and 390 *KB*/*s* respectively, while WSD and WSS algorithms are 380 *KB*/*s* and 375 *KB*/*s* respectively. The average segmentation speed of DD is 397 *KB*/*s*, which is higher than the 374 *KB*/*s*, 364 *KB*/*s*, and 372 *KB*/*s* of SP, WSD, and WSS algorithms. Based on the above results, the proposed DD algorithm has superior performance and performs well in text segmentation in IPORDM.

### 4.2 Performance analysis of HCRNN-AM

To verify the feasibility of HCRNN-AM in text sentiment classification, experiments were conducted on Weibo’s Emotional Analysis (WEA) dataset. The reason for choosing WEA dataset is that Weibo is currently the most free and active social platform for speech. Almost every college student’s mobile device has a Weibo app, which can to some extent reflect the current online public opinion atmosphere.

Table 2 shows Specific data of the sentiment category table in WEA dataset, with a total of 361784 sentiment annotation data. All data includes four emotions: joy, anger, disgust, and depression. There are 199512 joyful sentiment data, 51724 angry sentiment data, and 55274 disgust and anger sentiment data. The study first trained CNN on WEA dataset to verify its classification performance in short text. The control group selected Support Vector Machine (SVM) and K Nearest Neighbor (KNN) models. This experiment was conducted on Matlab simulation platform, where the convolutional kernel sizes of CNN were set to 3, 4, and 5, and the number of convolutional kernels was set to 128.

**Table 2:** WEA dataset emotion category table.

Emotion	Quantity/strip
Happy	199512
Angry	51724
Detest	55274
Depression	55274

**Fig 11:**
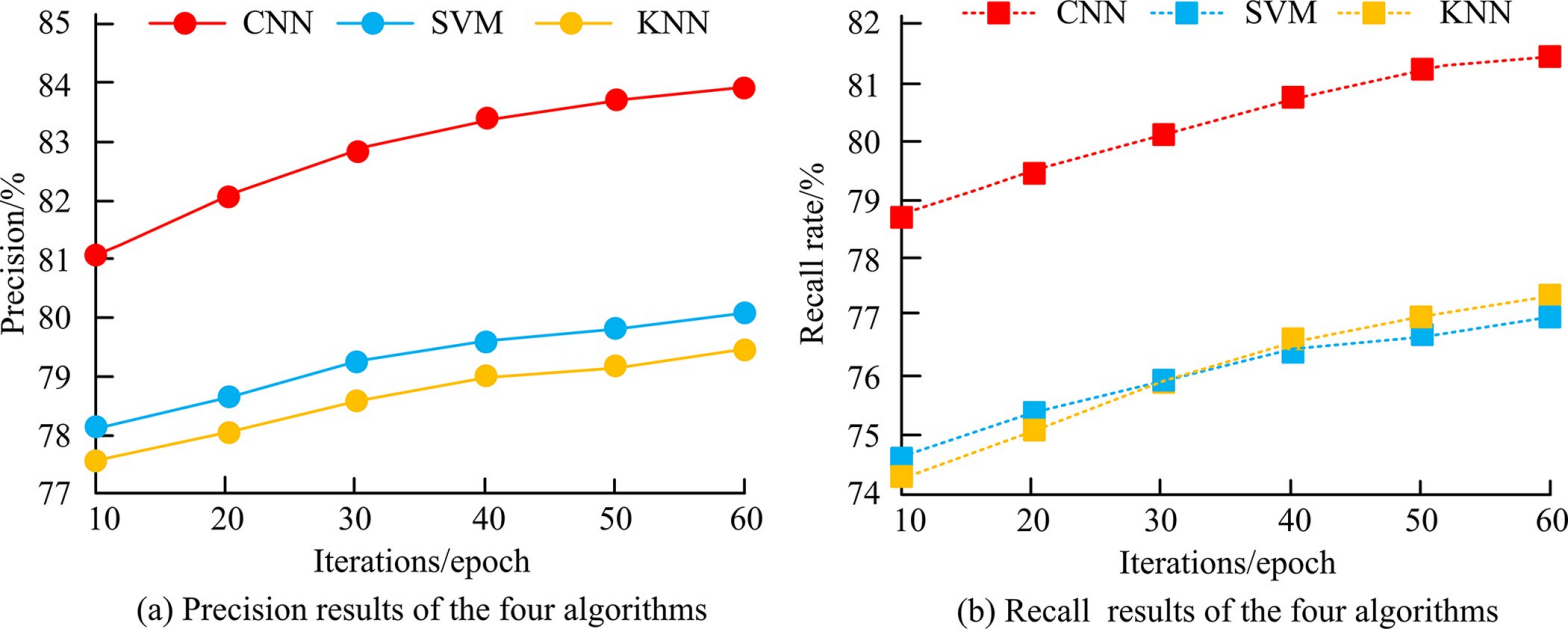
Precision and recall results of three algorithms.

Fig 11 shows the precision and recall results of the three algorithms. As iterations increase, the precision and recall of all three algorithms increase. Fig 11A shows the precision variation curves of the three algorithms. When the iteration is 10 epochs, the precision of CNN is 81.01%, the precision of SVM is 78.11%, and the precision of KNN is 77.73%. When the iteration is 60 epochs, the precision of CNN is 84.03%, while the precision of SVM and KNN is 79.78% and 78.89%. Fig 11B shows the recall rate variation curves of the three algorithms. When the iteration is 10 epochs, the recall rate of CNN is 78.87%, SVM is 76.21%, and KNN is 76.38%. When the iteration is 60 epochs, the recall rates of CNN, SVM, and KNN are 81.16%, 76.70%, and 76.97%, respectively. Based on Fig 11, CNN outperforms the other two algorithms, proving its good performance in short text classification. To further verify the classification performance of BLSTM model and BGRU in long text, SVM, KNN, and CNN were used for comparative experiments. The number of hidden nodes for BLSTM and BGRU was set to 256.

**Fig 12:**
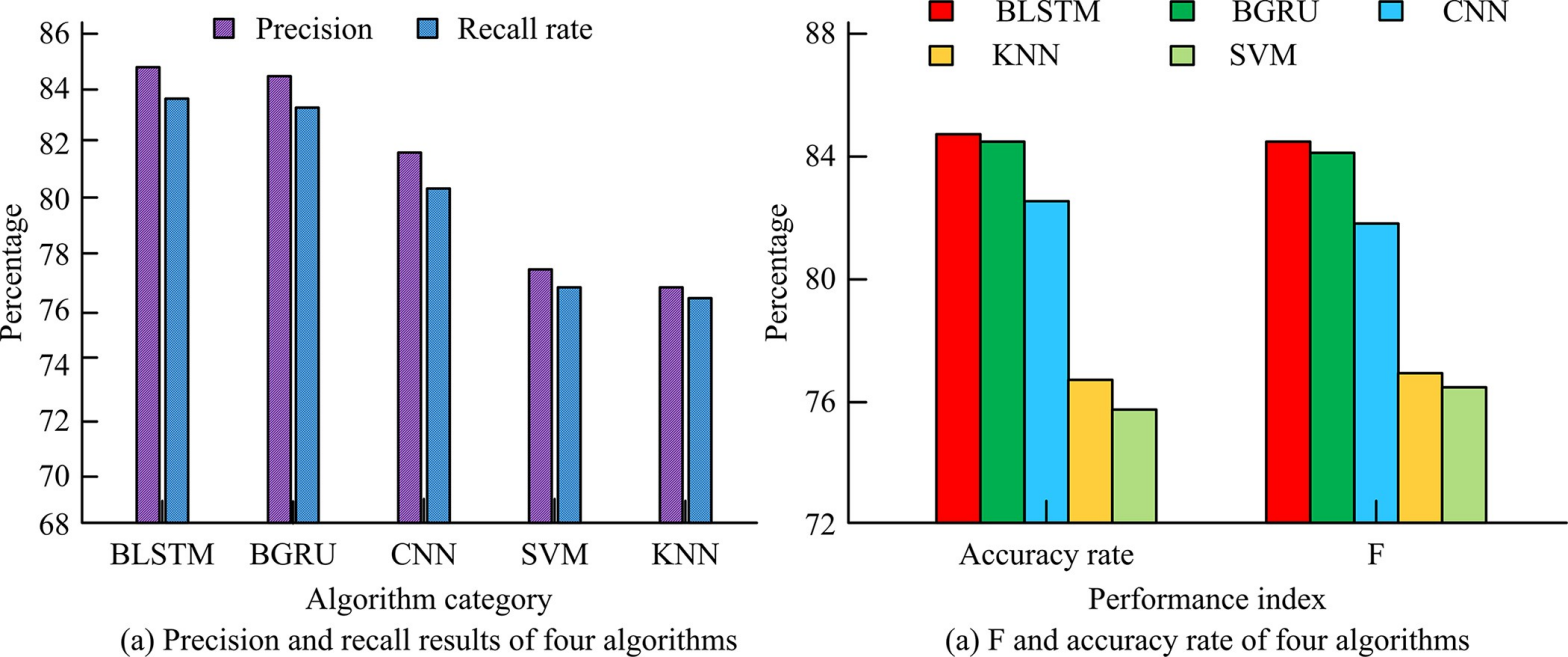
Evaluation index results of five algorithms.

Fig 12 shows the precision, recall, accuracy, and F-value results of five algorithms. Fig 12A shows the precision and recall results of the five algorithms. For precision, BLSTM is 84.95%, BGRU is 84.84%, CNN is 81.77%, SVM is 75.67%, and KNN is 74.87%. The recall rates of BLSTM and BGRU are 83.74% and 83.68%, respectively, while the recall rates of CNN, SVM, and KNN are 80.32%, 76.89%, and 76.73%, respectively. Fig 12B shows the accuracy and F-value results of five algorithms. For accuracy, BLSTM is 84.54%, BGRU is 84.33%, CNN is 83.13%, SVM is 76.41%, and KNN is 75.45%. F-values of BLSTM and BGRU were 84.48% and 84.42%, respectively, while F-values of CNN, SVM, and KNN were 82.13%, 76.21%, and 75.94%, respectively. To further validate the performance of HCRNN-AM based on CNN, BLSTM, and BGRU algorithms, comparative experiments were conducted using HCRNN-AM and End-to-end Trainable Neural Network (ETNN) models. AM dimension parameter in HCRNN-AM is 512, and the Learning rate and full connection layer node parameters are 0.01 and 256 respectively.

**Fig 13:**
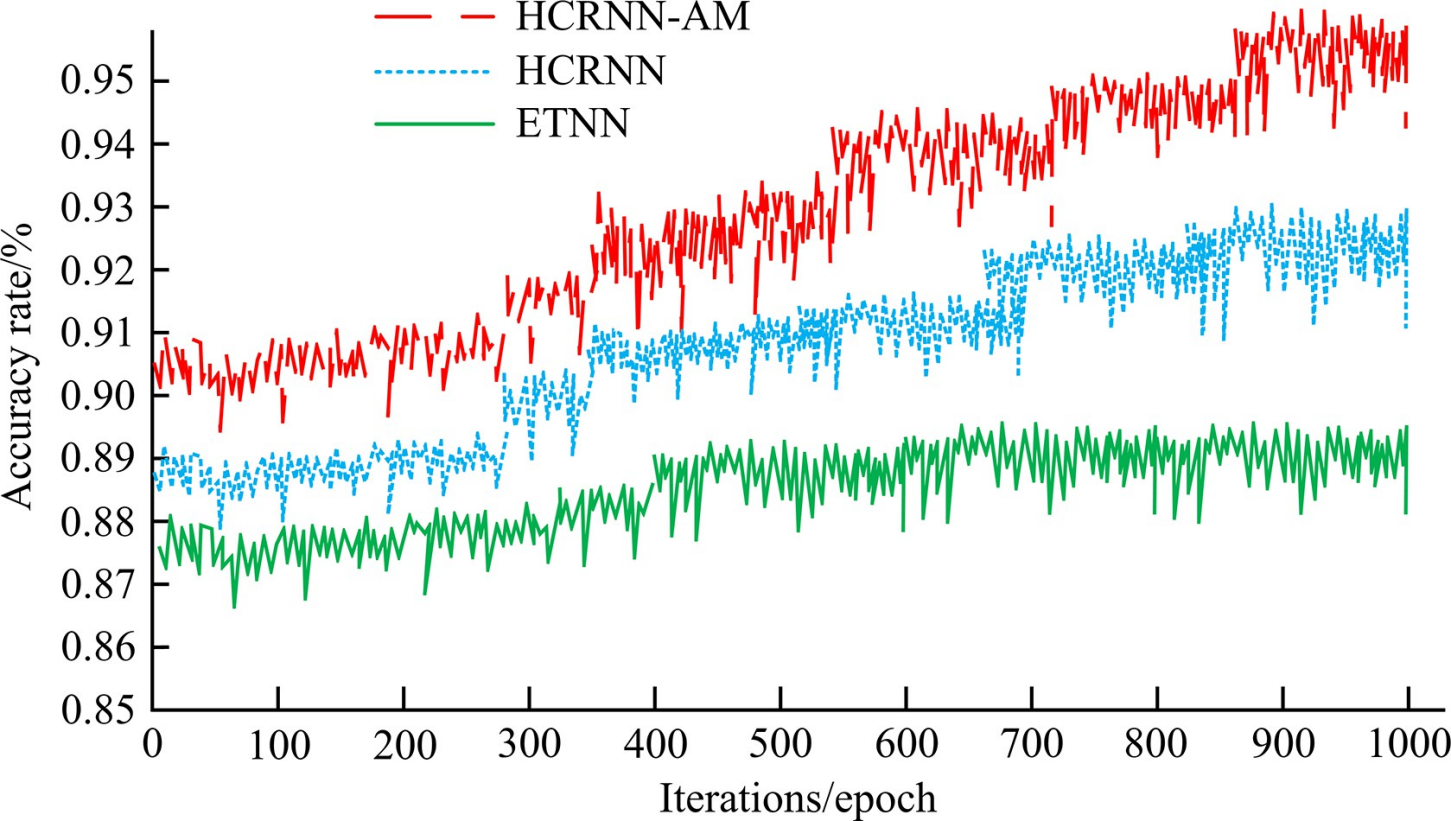
Accuracy curve of three models.

Fig 13 shows the accuracy variation curves of these three models. As iterations increase, the accuracy of all three models increases. For accuracy, at 1000 epochs of iteration, HCRNN-AM was 95.12%, HCRNN was 91.23%, and ETNN was 88.13%. Based on these above results, when HCRNN is added to am, its overall performance is greatly improved, and it can better perform text sentiment classification on social ipordm.

## 5. Conclusion

Campus social IPO monitoring is a complex task with multiple interdisciplinary and technical complexities. Traditional IPO monitoring often involves manual collection of IPOs, which typically requires a lot of manpower and material resources, and the search efficiency is not high. To address this issue, the study first utilizes DD algorithm for text segmentation, and then constructs HCRNN-AM based on CNN and RNN variant structures. These results indicate that the average precision of DD algorithm is 97.30%, which is 0.49% higher than SP algorithm. The average recall rate and F-value of DD are 79.55% and 87.53%, respectively, which are higher than SP’s 77.69% and 86.17%. In terms of word segmentation speed, DD is 397 *KB*/*s*, which is 23 *KB*/*s* faster than SP. In the classification test of long texts, the accuracy of BLSTM and BGRU were 84.54% and 84.33%, respectively, better than CNN’s 83.13%. F-values of BLSTM and BGRU were 84.48% and 84.42%, respectively, which were 2.35% and 2.29% higher than those of CNN. At 1000 epochs of iteration, the accuracy of HCRNN-AM was 95.12%, higher than 91.23% of HCRNN. In summary, the algorithm and model proposed in the study have robust performance and have performed well in the field of campus social IPORDM. However, there are still shortcomings in the research. As the HCRNN-AM based on DD algorithm needs to be applied to the RDM work of university public opinion, a system is also needed to provide a visual interface to help university personnel analyze IPOs. Further research will be conducted on this topic.
